# Quarterly Report of the Obstetric Practice in the Philadelphia Dispensary for the Seventh, Eighth, and Ninth Months, 1840

**Published:** 1840-09-26

**Authors:** Joseph Warrington

**Affiliations:** Accoucheur


*Quarterly Report of the Obstetric Practice in the Philadelphia Dispensary for the Seventh, Eighth, and Ninth months*, 1840. By Joseph Warrington, M. D., Accoucheur.

Twenty-two cases of labour at full term have been under care, besides one case of abortion at the sixth month of pregnancy.

Ten boys and eleven girls have been born at full time, one male child remaining undelivered at the death of the mother. The abortion was also a male child.

The average duration of labour in fifteen cases was thirteen hours, the extremes being four and twenty-four hours.

The average time required for the spontaneous delivery of the placenta in seventeen cases was twenty-eight minutes, the extremes being five and sixty minutes.

Of sixteen cases, of which the positions were carefully noted, fifteen were in the first, and one in the second of the vertex.

In one case the occiput became arrested at the superior strait, making it necessary to introduce the fingers to increase the flexion to enable it to descend.

In one case the anterior lip of the uterus remained so firmly stretched over the occiput as to retard delivery for several hours, and require the insinuation of the fingers to slip it over the occiput, and then to press upon the forehead to increase the flexion.

There were two cases of prolapsus of the cord,—one in which the parturient effort was so powerful and rapid in its progress as to expel the child before it could suffer much from compression. In the other, although the cord was several times reduced, the size of the head was so disproportioned to the capacity of the pelvis, that death, first of the child, and then of the mother, took place before delivery could be accomplished.

The forceps were applied in two cases,—one to assist in delivery from the inferior strait of a woman nearly exhausted by the fatigue of the labour, being in an advanced stage of phthisis, with a large cavity in the lungs; in the other instance to assist the delivery in the case in which the cord prolapsed, and could not be retained after it had been reduced.

There was one case of metritis, which occurred on the third day after a favourable delivery in a patient who resided in a damp, ill-ventilated cellar, the patient having sat upright in bed contrary to positive orders. She was treated actively by venesection, fomentations, purgatives, and vesication, and nearly recovered, when it became necessary to place her under the care of the guardians of the poor.

Two cases of prolapsus uteri occurred; one, in a patient who left her bed and walked into another room on the second day after delivery; the other had been subject to the affection previous to her last pregnancy.

The other woman did well.

One child was still-born, appearing to have been dead some time.

The rest of the children have done well.

